# Community Assets for Health Model and Assessment Scale: A Delphi-Based Analysis and Expert Validation

**DOI:** 10.3390/ijerph192113979

**Published:** 2022-10-27

**Authors:** Pablo Sáinz-Ruiz, José Ramón Martínez-Riera

**Affiliations:** Department of Community Nursing, Preventive Medicine, Public Health and History of Science, University of Alicante, E-03080 Alicante, Spain

**Keywords:** salutogenesis, health assets mapping, validation, Delphi technique, community assets for health assessment scale

## Abstract

The salutogenesis theory of Aaron Antonovsky and the Health Assets Model of Morgan and Ziglio have given rise to a notable interest in defining the resources available to individuals and the community to maintain or improve their health and well-being. The present study began by identifying the universal dimensions of Community Assets for Health, and then analyzed and validated an assessment scale following the Delphi method. A high degree of consensus was achieved among 13 experts from different disciplines. The results of the content analysis and statistical analysis led to a reconfiguring of an instrument that is so far unique in its approach. It is composed of 103 items across 14 dimensions (utility, intention, previous use, affordability, proximity, walkability, connectivity, intelligibility, identity, design, safety, diversity, public dimension, and sustainability).

## 1. Introduction

Many authors from a range of disciplines have attempted to define the resources that individuals and the community have at their disposal to maintain or improve their health and well-being. A notable contributor in the field of psychology is Antonovsky [1,2], with his theory of salutogenesis in which he defines the Sense of Coherence and Generalized Resistance Resources (GRRs). In the social sciences, Kretzmann and McKnight [3] imported the concept of community assets into their Asset-Based Community Development model (ABCD) and emphasized the community’s key role in identifying individual talents as well as a context’s environmental strengths or available resources [4].

Eriksson and Lindström [5] collected many converging concepts and theories under their salutogenic umbrella that constitute a positive approach to people’s health and quality of life. Such a positive health perspective is gaining ground with respect to the traditional biomedical line of action, which centers on deficits, treatment and prevention.

The definition of health assets advanced by Morgan and Ziglio [6] somewhat embraces all the above approaches, as they refer to “any factor (or resource), which enhances the ability of individuals, groups, communities, populations, social systems and/or institutions to maintain and sustain health and well-being and to help to reduce health inequities” (p. 18). This definition could be assimilated to that of Antonovsky’s GRRs, i.e., any characteristic, of any nature, genetic, biological, physical, material, cognitive, emotional, attitudinal, relational, sociocultural, spiritual or psychosocial of a person, group or environment that helps to manage stress effectively [1,2,7].

Incorporating Kretzmann and McKnight’s method into a more grounded conception of assets, the asset model for public health goes beyond intrapersonal means and incorporates any component that the community identifies as its own and as having the potential to improve coexistence, health, or to reduce the social inequities of health determinants [8].

This makes it difficult to fit the health assets concept into an operational definition that would facilitate the planning and executing of health-promoting interventions in the community. According to Stokols et al. [9], efforts should be prioritized by strategically relating resources to the stressors that matter. This way, synergies could be determined between the salutogenic approach and the deficit approach, between needs and assets, or between protective factors and risk factors, in the same way that Antonovsky referred to the health ease–disease continuum [10,11,12,13]. Assets gain meaning in the context of needs, and needs become significant in the quest for assets [3]. However, some authors, after reporting their assets mapping experiences [14,15], have highlighted the difficulties in defining the assets that influence population health the most, in determining “when a resource becomes GRR” [4] (p. 167) and why, as well as the values underlying such decisions or behaviors. This view can be somewhat linked to Antonovsky’s interrogation as to whether some GRRs would be more effective than others at addressing certain stressors [1].

A prior systematized review and content analysis allowed the authors of the present study to identify dimensions and characteristics that were “universally” related to the concept of community asset for health [16]. The in-depth search for scientific evidence and measuring instruments for each dimension resulted in an initial 14-dimensional instrument, encompassing 24 categories and including a total of 145 items. The tool was developed to answer the following questions: what is understood in the literature by health assets and what is not? What differentiates a community asset for health from other resources? Are all resources potential community assets for health? It therefore contributes to the instruments—long-awaited by researchers and technician-professionals—allowing adequately measuring and evaluating asset-based approaches [17].

This paper describes the process that focused on analyzing the Assessment Model of Community Assets for Health and on validating the initial instrument based on the opinions of experts from different disciplines.

## 2. Materials and Methods

### 2.1. Procedure and Framework

The present study was part of the doctoral work “Identification and Assessment of Health Assets: Epistemological Analysis and a Measurement Model” at the University of Alicante (Spain). The objective was to develop a method to validate and weight health assets. A systematized review as well as a content and inductive analysis of the dimensions that are universally identified as characteristics of community assets for health were conducted based on asset mapping research and experiences. The review and analysis centered on the questions Which? How? and Why? these community assets for health were selected.

First, a taxonomy of the dimensions was specified. The items enabling them to be analyzed were then determined. Thus, the first prototype of an assessment scale for community assets for health came into being, together with their weighting or prioritization.

The initial theoretical model and instrument (originally in Spanish) were then presented to a panel of experts following the Delphi methodology. The Delphi technique has amply proven to be a useful and flexible method for reaching consensus in an area of uncertainty or lack of empirical evidence [18]. The considerations that were agreed upon were unified and the instrument’s usefulness was validated.

### 2.2. Sample

Based on the range of participants recommended in the literature [18,19], the Delphi panel was made up of 13 experts (Table 1) together with an initial purposeful sample of 14 experts identified through convenience sampling based on the following criteria:

- Multidisciplinarity: a large number of experts had public health experience (*n* = 7; 54%); several were trained in nursing and other health sciences (*n* = 4; 31%), and others were experts in architecture, urbanism, anthropology and sociology (*n* = 1; 8%).
- Scientific/research or professional experience in the approach and methodology analyzed. Scientific experience (*n* = 13; 100%); professional experience (*n* = 10; 77%).
- Willingness to participate and commitment. The Delphi panel started during the summer period for a duration of 4 months.
- We also verified that they were familiar with the Delphi methodology and sufficiently mastered the electronic means of communication provided.

The participation rate was 100% during the initiation process, as well as in the first and second discussion phases (Q1 and Q2). The third phase (Q3) was an open discussion. The experts could freely reply within a given period of time, during which 7 experts gave complete answers (54%) and 3 partial replies (23%).

### 2.3. Phases of the Delphi Process

Each expert in the initial sample was contacted by telephone or by email and informed of the research topic together with its objectives, the purpose of the Delphi panel, the procedure guidelines and the estimated schedule. They were each asked to commit themselves fully throughout the process, which took place during the summer period (June–August 2020). The standard deadline for each discussion phase was 10 days. The second phase, however, was prolonged, as the experts’ initial responses were insufficient due to August being a holiday month. Three rounds of discussion were carried out (Q1–Q3), and the entire process lasted up to 76 days. The principles of iterativeness and feedback were consistently applied, and the results were presented in the form of thought syntheses and reached agreements [19].

The whole process was executed using the Google Form questionnaire model. A balance was sought between closed ended categorical answer questions and open questions so opinions could be freely expressed (Table 2).

- Phase Q1

In phase Q1, four general questions were asked in order to identify any dimension potentially missed.

The answers to the closed Q1 question were analyzed using descriptive statistics: frequencies and relative percentages of agreement with each dimension. A consensus was regarded as reached when 80% of the experts (10/13) were “at least in agreement” with the dimension (agree, quite agree, and totally agree), according to Landeta’s criterion [19] (p. 13).

In the case of the Q1 open answers, a content analysis was performed and the experts’ statements and contributions were taken into account. They were included in the analysis of the form that followed.

- Phase Q2

In the second discussion round (Q2), an in-depth and individualized analysis of the items initially included in the scale was performed in addition to the items proposed by the experts in the first round (145 + 2 items).

The experts were asked their opinion on the relevance of each item and whether they were adequately formulated or not. To measure the relevance, the MoSCoW method was used. It allowed collecting the experts’ opinions on the relevance of maintaining or suppressing the items, as well as the importance attributed to each of them (and therefore led to assessing the dimensions again, this time in a disaggregated way). Adequacy was measured by means of a closed question with a dichotomous answer (yes or no) and the option of adding comments/modifications in case of disagreement.

Based on the MoSCoW method [20], the assessments of each item were grouped in a dichotomous way: on the one hand, positive assessments, i.e., it was desirable or necessary to include the items; on the other hand, negative assessments, i.e., against maintaining the item (must have and should have vs. could have and won’t have). These responses were analyzed via descriptive statistics. The relevance of removing or maintaining the item was assessed according to the following criteria:

- Criterion for maintaining an item: at least 80% of the answers had to be *must have* and *should have*.
- Criteria for removing an item: (a) *won’t* responses had to exceed 20%; (b) *could have* answers had to exceed 60%; (c) the sum of *won’t have + could have* answers had to exceed 60%.

- Phase Q3

The third electronic submission invited the experts to again answer questions in a form. On the one hand, the form included questions on items for which no consensus had been reached regarding relevance or adequate wording. On the other, it included general questions about the instrument and the proposed model.

### 2.4. Data Analysis

The results of each phase of expert consultation were analyzed in relation to their content in the case of open questions of opinion and summarized statistically in the case of categorical questions. Descriptive statistical analyses were carried out using the SPSS v22 software (SPSS Inc., Chicago, IL, USA).

### 2.5. Ethical Considerations

The participants’ identity was kept anonymous. Data were anonymized and protected according to Spanish law (organic law 3/2018), and its European equivalent 2016/679.

All participants were informed of this study’s objectives and their informed consent was obtained prior to their participation. The authors declare that they have no conflict of interest or funding.

## 3. Results

### 3.1. Characteristics of the Experts

A total of 31% of the participants were women, i.e., 4 out of 13 experts. Some 92% (12 out of 13) had more than 10 years of research or practical experience in the field of Public Health from a positive social and health perspective (salutogenesis). Three had collaborated with the World Health Organization. Though they came from a range of disciplines, their lines of work were, overall, linked to the “Health in All Policies” approach and revolved around inequities in health, health promotion and sustainability, as well as community participation.

### 3.2. Dimensions and Modifications of the Instrument

An instrument composed of 14 dimensions, 24 categories and a total of 145 items was initially developed based on a systematic review and content analysis conducted previously [16], together with subsequent in-depth bibliographic reviews of each concept. In phase Q1, the experts proposed 2 new items and the final result of the Delphi panel finally reduced the instrument to a total of 103 items (−30% variation), maintaining the dimensions and categories (Table 3).

The analysis of the dimensions’ relevance did not reveal any substantial differences between the first assessment Q1 and the second examination Q2, the results in this second phase being the mean of the assessments of the items making up each dimension (Table 3). Thus, the “utility” dimension kept the best score, while the “public” dimension again received the worst, although some experts recognized the importance of the public nature of the resources due to their impact on social inequity reduction. The “sustainability” dimension ultimately received the second highest score after analyzing the items separately.

- **Utility**

An individual’s motivation to address a need appears the instant that need arises [21]. The significance and interest we give to resources derive from the usefulness we attribute to them according to our needs, values or culture. The latter are subjective: they are proper to an individual or a community. They do not constitute an inherent feature of the resource. However, the resource’s capacity to resolve one need or another, confer an added value to the resource, whether it be social, cultural or even environmental [22,23].

All the items in the “utility” dimension were assessed by the experts. The discussion focused essentially on two issues. The first was the relevance of including the basic needs mentioned in the initial model according to the functional patterns of Marjory Gordon and the hierarchy of Maslow. The second was that of considering the classification of Max-Neef, Elizalde and Hopenhayn [24], according to which “fundamental human needs” are finite, few and classifiable, and moreover, universal, i.e., they remain the same across all cultures and historical periods. The final instrument brings together these three models (Table 4) and proposes items for each of the seven needs.

- **Intention**

The Theory of Planned Behavior helps us to understand an individual’s behavioral process, particularly regarding the decision to use a resource [25,26]. According to the Theory of Planned Behavior, “intention” is the main motivator of behavior and, intention in turn, stems from Subjective Norm and Attitudes [25]. According to several authors [27,28], our motivation is greater when we perceive that such a behavior may be successful and when we have some internal control over this successful outcome.

After discussing the Subjective Norm concept, a disparity of opinions emerged as to whether or not the influence of social norms on the perception of health assets should be assessed.

*“… it is unclear whether the use of a resource for health reasons would go against social norms”*,

(Exp9)

*“… the resource must be used without having to be approved by anyone, it must be one’s own decision”*,

(Exp4)

*“… we know that not everyone behaves in accordance with social expectations. That is, transgression-in the strict sense of breaking a precept, a law … a social norm-can precisely be a factor that strengthens someone’s intention to use a certain resource”*.

(Exp6)

This divergence of opinion was observed in the assessments given to some items. One item referring to social norms met criterion (b) to be removed directly from the instrument but was reintroduced after a phase Q3 discussion because of its theoretical relevance. Its wording was modified (item 11, Appendix A-Table A1).

According to Wang et al. [26], attitude, previous use of a resource, and perceived accessibility also constitute antecedents of behavioral intent.

- **Previous use**

Various studies [27,28] as well as the content analysis of the systematized review conducted prior to the Delphi panel [16], highlight how a “previous use” of a resource influences its perception as a health asset or not.

This dimension and its items were not viewed negatively by the experts in any way, although the level of temporal disaggregation was proposed to be reduced from 4 to 3 items, taking into account the effect of frequency and currency of its previous use.

- **Accessibility (perceived): Affordability, proximity, walkability, connectivity and legibility**

Pirie (1979) had already noted that “accessibility is always created and is not just something to be had by virtue of one’s locale” (p. 307), in such a way that the model presented here extends the concept of accessibility to an individual’s subjective interpretation of the resource which depends on “affordability”, “connectivity”, “walkability”, and “intelligibility”, and not only proximity [26,29].

Thus, the proximity dimension was the worst rated in the accessibility dimension, compared to connectivity and walkability (Table 3). These dimensions reflect the findings of a major part of the literature that has succeeded in developing several indicators, such as Leslie’s walkability index [30,31,32,33,34], or quantitative standards of proximity [32,33,34,35,36,37,38,39] or connectivity [38,40,41,42,43,44]. The experts recommended avoiding technicalities in the case of several items in these dimensions, (e.g., items i32, i33, i34, i40, and i43) and reducing disaggregation (e.g., items i30–31, i32–34, and i52–56). In the case of proximity, items were reduced from 4 to 2 items (f26 and f27) and in the case of intelligibility, from 9 to 6 items (f37–42).

In the affordability dimension, item i27 (Table 5) was positively evaluated by most experts but questioned by two of them due to it representing a sensitive and self-declared statement, that could prove to be unfruitful. The modification of its wording in simpler and less specific terms (item f23) was regarded as positive by all experts in Q3. Moreover, the items initially proposed to refer to the economic aspect of perceived accessibility (i30, i31) were synthesized in a single item (f25).

- **Identity**

The individual or social “identity” represented by the resource. This identity contemplates the subjective manifestation of a resource’s historical, cultural or social value for individuals or the community. The current model analyses this dimension through three categories extrapolated from Lalli [45] and Thomas. It was positively assessed by the experts: singularity (86%), appropriability (81%) and attachment (77%).

Some items were considered repetitive in the individual item assessment, thus illustrating the complex conceptual distinction between categories. For example, item i58 “The resource or heritage is perceived as characteristic of the community” (85%) in the singularity category, and itemi70 “The resource is perceived as proper to the community” (92%) in the attachment category were perceived as repetitive.

Experts recommended the use of other concepts in the wording of some items:

*“I would eliminate ‘heritage’, it is not easy to understand”*;

(Exp4)

*“People may find it hard to understand the expression singular/distinctive”*;

(Exp9)

*“I do not see it as essential, and it may not be fully understood either”*.

(Exp12)

- **Design**

The “design” dimension was the most extensive in the instrument’s initial configuration and included the notions of configuration, functionality and comfort, maintaining a parallelism with the principles proposed by Vitruvius: venustas, utilitas, and firmitas, in accordance with the organization of the items based on the Design Quality Indicator scale [22].

This dimension was not the worst valued generally, but some of its items were (phase Q2). Three items were removed directly from the instrument because they met the *won’t have* negative assessment criterion: items i74, i75, and i97 (Table 6). In addition, the items relating to comfort (thermal, visual and air quality) were re-formulated so as to include proposals to reduce the disaggregation level: items i92–95 into f65, i96–99 into f66; items i100–102 into f67, and items i103–105 into f68. Other items were considered repetitive by the experts and were unified: items i76 and i82 into f56, items i80 and i81 into f58, items i83 and i84 into f60, and items i88 and i89 into f63. The modifications were discussed again in phase Q3 and were well received:

*“I agree with the analysis performed and its implications”*.

(Exp1)

- **Safety (perceived and objective)**

Based on the content analysis of the reviewed literature [16], the concept of “safety” was understood to be shaped through a subjective perception, whether individual or collective, and through objective measurements. The data support the fact that people living in safe and friendly environments can be more active and make greater use of resources [46,47].

The dimension was well valued in both Q1 (100%) and Q2 (75%), but item i120 (“Factors such as age or sex, ethnicity or religion, or disability, do not affect the perception of safety in the resource or its environment”) gave rise to debate due to discrepancies in the *won’t have and must have* assessments (0.84/1), as well as in the comments referring to the item’s disaggregation:

*“… I would separate it”*,

(Exp2)

*“it is not easy to understand, we would have to differentiate the different factors to know which factor they are linking to the perception of safety”*.

(Exp5)

- **Diversity**

The dimension of “diversity” referred to the idea of quantity from the external perspective of the territory, and variety from the internal perspective of the resource, its range of products [48] or equipment on offer that filled the same function. The dimension included three items. The second, “The resource is scarce” was removed from the final instrument with 62% positive ratings but two *won’t have* responses. Experts also encouraged simplifying the wording of the other two (items f82 and f83, Appendix A).

- **Public**

Undoubtedly, the “public” dimension was the worst valued, both in the first discussion round and in later ones, reaching 59% of agreement on relevance in the analysis of aggregate items (f84–86).

On an individual basis, none of the three proposed items were truly positively valued, whether in the category referring to the exclusivity and rivalry factors that favored inequalities of access, or in the “privacy” category.

In phase Q3, these items were raised again for discussion. No consensus was reached, but some favorable views were expressed:

*“Indeed, the perception of exclusivity and/or rivalry can influence the assessment of a resource as an asset”*.

(Exp7)

- **Sustainability**

The sustainability dimension included a large number of concepts, such as the asset’s resilience over time [49], its intersectorality or centrality in the territory [50] and/or participation in the community, and other values such as the reduction in social inequities [15,51,52] and environmental sustainability.

Unlike the previous dimensions, “sustainability” was the only one to have received a better evaluation after its items were assessed separately (Q2). The worst valued item was the one referring to private or social profitability, fulfilling criterion (a) of negative assessments. The issue of an ordinary citizen’s difficulty in measuring this dimension was again raised. For other items, the wording was simplified, technicalities were removed, and four items were deleted.

### 3.3. Measurement of the Instrument

If the instrument is used as a two-option response checklist, the scores at the scale extremities would match the number of items answered affirmatively: from 0 points to 103. In our case, the experts recommended a positive, five-point scoring system:

*“I would recommend a Likert-5 scale and only positive scores, from 1 to 5 points”*.

(Exp4)

In this case, the scores would be a minimum of 103 points and a maximum of 515 points. The final score, organized into three ranges, would be as follows: 103–240, 241–377; 378–515.

In some studies, such as that of Mosavel, Gough, and Ferrell [53], the asset mapping process differentiates between health assets and potential resources. Based on this latter proposition, range-based scores could be orientative regarding the distinction between a potential resource and a community asset for health. In this sense, we advanced the following proposal: the first score range would correspond to that of a mere resource without any major health-related significance (103–240 points); the intermediate range (241–377 points) would refer to a potentially significant resource regarding the maintenance or improvement of health; and the higher range to that of a community asset for health (378–515 points).

## 4. Discussion

To effectively undertake any strategic action within a community—involving the connecting and mobilizing of resources and support networks existing in the territory [54,55,56]—an initial step of identification, mapping and assessment of community assets for health must be carried out. This poses certain difficulties to technicians and citizens. A major difficulty reported in the literature is that of reaching an agreement on what a territory’s community assets for health actually are and why [14,15]. While a large number of studies have examined individual personal assets, and psycho-social strengths, few have focused on physical, material, and community resources [57,58].

The present work proposes an instrument of a unique nature to date: it serves as a citizenship guide on the perceptual and objective components of relevant community assets for health, supported and highlighted by extensive studies in different disciplines (from social and environmental psychology to ecology, urbanism or economics). These works refer to specific contexts, such as parks or public areas [59,60], or focus punctually on some of the 14 dimensions, such as accessibility [61], walkability [62], design [23], or sustainability [63]. Yet, no other study has hitherto proposed indicators based on a comprehensive approach to individuals’ psychological-behavioral approaches to their environment and a positive view of health.

Our proposal centers in particular on the identification, assessment and dynamization of community assets for health. The initial assumption was that not all a territory’s resources can be identified as assets, nor are all assets—identified as such—considered equally relevant when they are mobilized in community health improvement strategies. The model proposed in this study is based on the following premise: regarding individual or collective perceptions, the three components of Antonovsky’s Sense of Coherence [1,2] influence how a resource is identified as an asset—among all those in a territory, i.e., when an asset is acknowledged (understood), managed and perceived as playing a significant role in maintaining or improving health. In this way, a resource’s availability does not imply that it is recognized as an asset, and even if it has been identified by the individual or group, it will not necessarily be perceived as valuable or significant.

The instrument allows to assess the resources “utility” considering the fundamental needs that give them significance, that is, recognizing the synergies between the salutogenic approach and that of deficits, between needs and assets [11,12,13]. However, the instrument also introduces other aspects that are closely related to significance, such as the dimension of “intention to use” or “identity”. The importance of significance has been mentioned by several authors [13,15]. It emerged in the expert panel and these dimensions were the best valued. So were the dimension and items of “sustainability”. Moreover, authors such as Flint [64] agree on the interrelation of sustainability with health at all organizational levels, from the maintenance or durability of the resource to social and environmental sustainability.

On the other hand, other dimensions failed to reach a significant consensus among experts although the literature considered them relevant, such as: the “design” of the resource; its flexibility [65,66] or aesthetics [67,68]; and the conditions of financial [15,47,48] and “public” affordability [69]. For this reason, modifications were made and the items were finally accepted by most experts.

These dimensions and categories are organized in a double-entry diagram that sorts the different variables, categorizing a resource as a community asset for health based on two criteria: the horizontal axis places the variables according to their more or less close links with the concepts of significance, intelligibility and manageability [1,2]; and the vertical axis allows to place these same variables according to whether they correspond to the resource’s internal (usually objective) factors or attributes, or to external factors that are more related to an individual or community viewpoint (subjective). (Appendix B-Figure A1).

The idea of the “resource–community asset/for health” continuum could include the so-called potential assets that some authors have distinguished from primary assets [14,53,54] and that the authors propose here according to the instrument’s scoring ranges.

### Implications for Research and Practice

The proposed model allows advancing in the epistemological and methodological disquisition concerning the broad salutogenic approach, which is based on protective factors and health promoters, as well as synergies with the needs-oriented biomedical paradigm.

Following the questions raised by the expert panel, it would seem pertinent to further examine the weight of the different dimensions in the final assessment of community assets for health. Although most experts considered that all items and their dimensions should have the same weight, two suggested the opposite:

*“I would weight some dimensions more than others, […] in my opinion, the most significant items are those relating to identity and sustainability”*.

(Exp4)

The multiple responses regarding this scale and in different contexts make it desirable to pursue research in this direction, taking advantage of GIS technologies and network analysis methods [70].

In addition, enabling the instrument to be loaded into a computer program or mobile application would contribute to realizing the authors’ practical vision of the instrument, and to advance in “collaborative mapping”, in accordance with Sajja and Akerkar [71] (p. 2). The community could, in this way, be empowered with respect to its own health, free access could be democratized to all, and geostatistical analyses of resources according to the population needs could be conducted.

## 5. Conclusions

This study provides a model for the assessment and weighting of community assets for health. The final instrument resulted from a consensus reached among experts in public health and other disciplines.

A prior comprehensive systematized literature review and content analysis of experiences in health asset mapping [16] were the starting point in establishing the model’s dimensions and indicators. The study led to a Community Assets for Health Assessment Scale and Model, which was positively valued by experts and achieved a high level of consensus (83% on average). The instrument serves as a guide to reflect on the qualities that differentiate a community resource from a community asset for health. To date, no other instrument based on the salutogenic approach has proposed a list of criteria to guide the discussion, measurement and weighting of mapped resources. Necessary tools to carry out health diagnoses of territories that guide the planning of health actions.

This model contributes to the necessary promotion of a method focused on salutogenesis in all policies and based on measurable and verifiable criteria. It aims to complement the necessary triangulation procedures of diverse opinions and perspectives, guaranteeing the principles of equity and community participation in health issues.

In the proposed model, Aaron Antonovsky’s salutogenesis theory converges with the assets mapping method and, for the first time, fundamental human needs are interrelated with other dimensions that refer to an asset’s significance for the individual (utility, intention, previous use, and even the identity dimension), as well as health equity indicators and other determinants. It also highlights the resource’s qualities with respect to its environment (diversity, connectivity or intelligibility, among others), its centrality in the community network, and the attention paid to reducing health inequities.

The credibility and transferability of the model and instrument did undergo a qualitative examination. Nevertheless, this study’s main limitation is that no empirical verification and statistical validation were performed. The instrument was initially drawn up based on a comprehensive literature review of asset mapping experiences. In addition, its validity was reaffirmed by the high consensus that was achieved among experts from different disciplines and on different geographical, cultural and sociodemographic environments. Nevertheless, it would be relevant to test the instrument and its application empirically in different contexts. A factor analysis could lead to proposing a shorter scale, which would make it easier to use by communities and institutions.

## Figures and Tables

**Table 1:** Profiles of the Delphi panel experts.

Expert Code	Discipline	Experience (Years)	Manager	Research	Line of Work *
Exp1	Public Health	>10	Yes	Yes	WHO. Policies in HP
Exp2	Public Health	>10	Yes	Yes	Inequities and local action; IAP
Exp3	Sociology	>10	No	Yes	Citizen participation; IAP
Exp4	Health Sciences	>10	No	Yes	HA approach
Exp5	Architecture	7	Yes	Yes	Strategic design and participation; Inequities
Exp6	Anthropology	>10	Yes	Yes	WHO; inequities in health and HA approach
Exp7	Public Health	>10	Yes	Yes	WHO; healthy cities and health services
Exp8	Education and Public Health	>10	Yes	Yes	HP in childhood-adolescence and HA approach
Exp9	Infirmary	>10	Yes	Yes	Health management and health care approach
Exp10	Nursing and Public Health	>10	Yes	Yes	Health Promoting Universities. HE
Exp11	Town Planning	>10	Yes	Yes	Sustainable urban development and transport systems
Exp12	Nursing and Public Health	>10	No	Yes	HP and inequities in health
Exp13	Anthropology	>10	Yes	Yes	HP and health inequities; HA approach

* WHO = World Health Organization; HP = health promotion; IAP = action-participatory research; HA = health assets; HE = health education.

**Table 2:** Delphi panel questions (Q1–Q3).

Questions	Open	Closed
Q1	What other dimensions do you believe are decisive for a universal identification and assessment of a resource as a community asset for health? Do you know any validated measurement scales for the dimension(s) you have proposed?	Of the 14 dimensions initially contemplated according to the literature review, indicate your level of agreement regarding the relevance of using these dimensions to define a resource as a health asset ^a^
Q2	How appropriate do you consider the item is to measure the dimension? ^b^	Do you consider the wording of the item appropriate? ^c^ If not, how would you re-formulate it?
Q3	When scoring the “items” would you give the same value to each of them? If not, which items do you regard as the most important?… Would some “dimensions” weigh more than others?	Assessing the instrument as a whole, and based on the previous rounds of discussion, do you believe any dimension or categories described should be included or re-formulated? ^c^

^a^ The 7-point Likert responses: strongly disagree, rather disagree, disagree, neither agree nor disagree, agree, rather agree, and strongly agree. ^b^ For each item. Answers based on the MoSCoW method (must have, should have, could have and won’t have). ^c^ Dichotomous answer: yes or no.

**Table 3:** Dimensions, categories and number of items in the final instrument. Consensus and variation before and after the Delphi panel.

Dimension	Categories	Item Numbering ^a^	Compliance Q1 ^b^	Compliance Q2 ^c^	Items Pre-Q1 ^d^	Items Post-Q2 ^e^	Pre–Post Variation
*Utility*	*-*	1–9	100%	92%	11	9	−18%
*Intention*	*Subjective Norm, Attitude, Motivation*	10–19	85%	60%	11	10	−9%
*Previous use*	*-*	20–22	92%	81%	4	3	−25%
*Affordability*	*Circumstances, Opportunity, Economic Accessibility*	23–25	92%	75%	4	3	−25%
*Proximity*	*-*	26–27	100%	65%	4	2	−50%
*Walkability*	*Rectitude, Integrity*	28–34	85%	81%	8	7	−13%
*Connectivity*	*-*	35–36	85%	81%	4	2	−50%
*Intelligibility*	*Visibility, Transparency/Clarity*	37–42	85%	77%	9	6	−33%
*Identity*	*Singularity, Appropriability, Attachment*	43–52	100%	82%	14	10	−29%
*Design*	*Configuration, Funcionality, Comfort*	53–68	85%	74%	35	16	−45%
*Safety*	*Safety (perceived), Security (objective)*	69–81	100%	74%	16	13	−19%
*Diversity*	*-*	82–83	85%	72%	3	2	−33%
*Public*	*Public, Privacy*	84–86	77%	59%	3	3	0%
*Sustainability*	*Durability, Economic and Environmental Sustainability, Centrality, Equity/Inclusiveness*	87–103	85%	87%	21	17	−19%
**Total/Mean:**			90%	76%	147	103	−30%

^a^ Question numbers in the final instrument including 103 items. ^b^ Share (%) of positive assessments of the 7-point Likert answers: responses: agree, quite agree, and strongly agree. ^c^ Average share (%) of positive assessments: should have and must have. ^d^ Number of items included in the instrument prior to phase Q1. ^e^ Number of items included in the instrument after phase Q2 and agreed upon in phase Q3.

**Table 4:** Basic human needs according to Abraham Maslow, Manfred Max-Neef and Marjory Gordon, and contribution of the model.

Abraham Maslow	Manfred Max-Neef	Marjory Gordon	Final Model
Physiological	Subsistence	Nutritional	Subsistence
		Elimination	
		Sleep and rest	
Safety	Protection	Safety	Protection
Social	Affect	Role and relationships	Role and relationships
	Participation	Sexuality and reproduction	
	Understanding	Cognitive and perceptual	Understanding
Esteem	Leisure	Activity and exercise	Leisure
	Identity	Self-perception	Self-perception ^a^
Self-realization	Freedom	Values and beliefs	Self-realization ^a^
	Creation		

^a^ The needs of self-perception and self-realization share notable similarities that become evident when the three theoretical models are analyzed.

**Table 5:** Notable modifications of items of (perceived) accessibility.

Dimension	Initial Writing and Item Number/s (iN°)	Final Drafting and n° Item/s (fN°)
Affordability	Item i27—I am physically, intellectually and emotionally able to make use of the resource, or to participate in the activity.	Item f23—I can make use of the resource.
	Item i30—Making use of the resource, participating in the activities or enjoying the services, has no financial cost. Item i31—I have the necessary economic means to access and make proper use of the resource and/or participate in the activity, without this implying my renouncing other more important alternatives.	Item f25—The use of the resource has no financial cost or is acceptable.
Intelligibility	Item i52—The information is recognizable (distinguishable) and adaptable to users. Item i53—The information and operation of the user interface is readable and intuitive (they are understandable). Item i54—User interface and navigation components facilitate interaction (are operable). Item i56—The content is intelligible enough for it to be reliably interpreted by a wide variety of users.	Item f42—The content is sufficiently clear for it to be reliably understood by a wide range of users.
Proximity	Item i32—The resource is located in the community within 4500 m (or 60 min on foot) in the road network buffer. Item i33—[…] in the neighbourhood less than 800 m away (or 10 min on foot). Item i34—[…] less than 300 m (5 min on foot).	Item f26—The resource is near on foot: 60, 30, and 5 min.
Walkability	Item i40—The slope of the street does not make it difficult to go on foot (a slope of less than 5%).	Item f31—The street slope does not make it difficult to go on foot.
	Item i43—The environment of access to the resource is friendly and safe, and the urban compactness is proportionally adequate (sky view opening between 36–72°).	Item f34—The environment of access to the resource is friendly (it is spacious and you can see the sky).

**Table 6:** Items in the “design” dimension before and after the expert panel.

Initial Wording and Item Number (iN°)	Final Wording and Item Number (fN°)
i71—The building’s structure is efficient and makes a maximum possible use of the available space.	f53—*The design of the resource facilitates its functioning.*
i72—The resource is well designed/organized allowing the total population to make use of it (universal design).	f54—The resource is well organized, allowing the total population to make use of it (universal design).
i73—The resource is sufficiently spacious for the usage or services for which it is intended.	f55—The resource is sufficiently spacious for the usage or services for which it is intended.
i74—The resource takes advantage of its orientation on the site.	*Item removed from the final scale*
i75—The resource responds to the environment’s microclimate.	*Item removed from the final scale*
i76—The resource’s infrastructure is sufficient (facilities, objects, materials).	f56—*The resource’s infrastructure is sufficient (facilities, material means…)*.
i82—The resource’s facilities are adequate enough to meet the objective functions.	
i77—Elements that evoke nature (visual perception of green greater than 20% of the resource’s total space) can be observed.	f57—Elements inspired by natural spaces can be observed (visual perception of green greater than 20% of the resource’s total space).
i78—The form and elements used are well detailed or precisely chosen.	*Item removed from the final scale*
i81—This resource is generally attractive.	f58—This resource is generally attractive.
i80—The resource produces a good first impression.	
i79—The elements used (colour, textures, flora, etc.) improve the pleasure of use of the resource.	f59—*The decoration is attractive (colour, textures,.. they make the resource more pleasant to use)*.
i83—The configuration of the resource makes it adaptable to changes in usage.	f60—*The resource is easily adaptable to different uses.*
i84—The resource has a modular infrastructure that promotes the constant and optimal use of space.	
i85—The resource can be adapted to develop different functions non-simultaneously.	
i86—The resource services are available over generous opening hours or are adapted to the specific needs of its users.	f61—The resource services are available over generous opening hours or are adapted to the specific needs of its users.
i87—The resource simultaneously offers different opportunities or functions.	f62—*The resource offers several services or functions at the same time.*
i88—The resource design is practical, pleasant or relaxing.	f63—*The resource design is pleasant or relaxing and users feel comfortable.*
i89—The resource generates a low number of complaints by users.	
i90—The resource and its facilities generally look properly maintained.	*Item removed from the final scale*
i91—The resource looks cared for, clean and tidy.	f64—The resource looks cared for, clean and tidy.
i92—Acoustic quality is appropriate for use and comfort	f65—*The resource is calm, exposure to noise is low, or quiet areas are made available.*
i93—The resource is calm or provides areas that convey peace.	
i94—The resource does not permit a noise exposure level over 55 dBA between 7 a.m. and 10 a.m.	
i95—The resource does not permit a noise exposure level over 35 dBA during the night.	
i96—Air quality is appropriate for the use and pleasant experience of the resource.	f66—*Adequate air is breathed and the smells are pleasant.*
i97—External air quality is adequate.	
i98—You can breathe a fresh and pleasant atmosphere.	
i99—The smells or fragrances in the space are pleasant.	
i100—The resource’s ambient temperature is suitable for use (technically 20 to 26 °C, or an energy balance of de −50 y 50 W/m^2^).	f67—*The resource’s ambient temperature is suitable for use.*
i101—The resource’s ambient temperature is adequate for more than 8 h a day in summer.	
i102—The ambient temperature of the resource is adequate for more than 4 h a day in winter.	
i103—The resource is suitable regarding its lighting and chosen colours.	f68—*There is enough natural light and the lighting is adequate.*
i104—There is enough natural light.	
i105—There is sufficient artificial lighting in the resource.	

**Figure A1:**
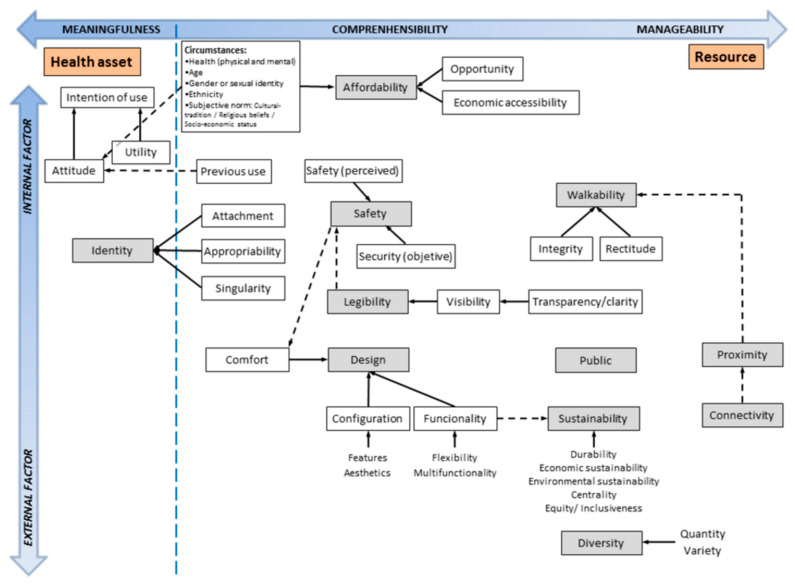
Model for the Evaluation of Community Assets for Health, interrelation of the dimensions and categories.

## Data Availability

The data are presented in this paper. The Community Assets for Health Assessment Scale can be consulted in full and in Spanish in Appendix A and the theoretical model in Appendix B.

## Appendix A

**Table A1:** Community assets for health assessment scale.

Items		Answers * 1 2 3 4 5
1	El recurso satisface directa o indirectamente la necesidad de subsistencia (alimentación, descanso, trabajo, vestimenta…)	☐ ☐ ☐ ☐ ☐
2	El recurso satisface directa o indirectamente la necesidad de protección (seguridad)	☐ ☐ ☐ ☐ ☐
3	El recurso satisface directa o indirectamente la necesidad de rol y relaciones (afecto, relaciones sociales, amistad, participación,…).	☐ ☐ ☐ ☐ ☐
4	El recurso satisface directa o indirectamente la necesidad del entendimiento (conocimiento, estudio, meditación,…).	☐ ☐ ☐ ☐ ☐
5	El recurso satisface directa o indirectamente la necesidad del ocio (actividad física, diversión, relajación, juego,…).	☐ ☐ ☐ ☐ ☐
6	El recurso satisface directa o indirectamente la necesidad de autopercepción (identidad).	☐ ☐ ☐ ☐ ☐
7	El recurso satisface directa o indirectamente la necesidad de autorrealización (valores y creencias, libertad, creación).	☐ ☐ ☐ ☐ ☐
8	El recurso es importante para la salud o bienestar personal.	☐ ☐ ☐ ☐ ☐
9	El recurso contribuye al mantenimiento o mejora de la salud o bienestar de la comunidad (familia y terceras personas).	☐ ☐ ☐ ☐ ☐
10	La gente que es importante para mí (familiares y/o amigos) aprueban que haga uso del recurso.	☐ ☐ ☐ ☐ ☐
11	Hacer uso del recurso está bien visto.	☐ ☐ ☐ ☐ ☐
12	Creo que hacer uso del recurso me resultará «totalmente útil—totalmente inútil».	☐ ☐ ☐ ☐ ☐
13	Creo que hacer uso del recurso me resultará «totalmente eficaz—totalmente ineficaz».	☐ ☐ ☐ ☐ ☐
14	Creo que hacer uso del recurso me resultará «totalmente ventajoso—totalmente perjudicial».	☐ ☐ ☐ ☐ ☐
15	Creo que hacer uso del recurso me resultará «totalmente inteligente—totalmente estúpido».	☐ ☐ ☐ ☐ ☐
16	Creo que hacer uso del recurso me resultará «totalmente agradable—totalmente desagradable».	☐ ☐ ☐ ☐ ☐
17	Hacer uso del recurso conducirá al resultado esperado.	☐ ☐ ☐ ☐ ☐
18	Tengo intención de hacer uso del recurso en los próximos meses.	☐ ☐ ☐ ☐ ☐
19	Tengo intención de hacer uso del recurso en los próximos años.	☐ ☐ ☐ ☐ ☐
20	El recurso se ha utilizado en los últimos meses.	☐ ☐ ☐ ☐ ☐
21	El recurso se ha utilizado en los últimos años.	☐ ☐ ☐ ☐ ☐
22	El recurso se utiliza de forma periódica.	☐ ☐ ☐ ☐ ☐
23	Puedo hacer uso del recurso.	☐ ☐ ☐ ☐ ☐
24	Dispongo del tiempo necesario para hacer uso adecuadamente del recurso sin renunciar a otras alternativas igualmente importantes.	☐ ☐ ☐ ☐ ☐
25	El uso del recurso no tiene coste económico, o es asumible.	☐ ☐ ☐ ☐ ☐
26	El recurso está próximo caminando a pie: 60 min–30 min–5 min.	☐ ☐ ☐ ☐ ☐
27	El recurso es visible en el entorno de mi actividad diaria, trabajo, ocio…	☐ ☐ ☐ ☐ ☐
28	Las personas pueden caminar fácilmente hacia el recurso.	☐ ☐ ☐ ☐ ☐
29	El recurso tiene buena comunicación a pie con otros recursos dentro de la zona.	☐ ☐ ☐ ☐ ☐
30	No hay barreras físicas de la configuración urbana (grandes calles, autopistas, vallas/muros…) o accidentes geográficos (lago o río, terreno escarpado) que dificulten realizarla a pie.	☐ ☐ ☐ ☐ ☐
31	La pendiente de la calle no dificulta realizar la ruta a pie.	☐ ☐ ☐ ☐ ☐
32	El diseño de la calle ayuda a realizar la ruta a pie (anchura de la acera).	☐ ☐ ☐ ☐ ☐
33	El trayecto resulta atractivo para el peatón por la diversidad de usos de la calle (comercial, ocio…).	☐ ☐ ☐ ☐ ☐
34	El entorno de acceso al recurso es amigable (espacioso y con cielo visible).	☐ ☐ ☐ ☐ ☐
35	Este recurso está bien conectado con otros puntos de interés de la ciudad.	☐ ☐ ☐ ☐ ☐
36	Para llegar al recurso, se puede acceder a una variedad de opciones de transporte a menos de 5 min a pie (autobús, tranvía, metro, automóvil,…).	☐ ☐ ☐ ☐ ☐
37	El recurso es distinguible o notorio en el territorio.	☐ ☐ ☐ ☐ ☐
38	Los accesos al lugar son claros y visibles (perceptibles para cualquier persona).	☐ ☐ ☐ ☐ ☐
39	Dispone de algún medio informativo (página web, tablón de anuncios,…) a través del cual sea posible acceder o se pueda solicitar dicha información.	☐ ☐ ☐ ☐ ☐
40	Si el recurso dispone de medio informativo… Está configurado de tal forma que garantiza una legibilidad universal (respondiendo a cualquier limitación: visual, auditiva, cognitiva, cultural-lingüística,…).	☐ ☐ ☐ ☐ ☐
41	Si el recurso dispone de medio informativo… Se puede acceder al contenido desde diferentes dispositivos.	☐ ☐ ☐ ☐ ☐
42	Si el recurso dispone de medio informativo… El contenido es lo suficientemente inequívoco como para que pueda ser interpretado de manera confiable por una amplia variedad de usuarios.	☐ ☐ ☐ ☐ ☐
43	El recurso es especialmente significativo para la mejora de mi salud o bienestar.	☐ ☐ ☐ ☐ ☐
44	El recurso es percibido como característico de la comunidad.	☐ ☐ ☐ ☐ ☐
45	Se trata de un recurso que tiene elementos singulares/distintivos.	☐ ☐ ☐ ☐ ☐
46	Los rasgos característicos del recurso (factor humano, servicios que ofrece) son difícilmente imitables o replicables.	☐ ☐ ☐ ☐ ☐
47	He tenido tantas experiencias de uso del lugar o recurso que me siento relacionado con él.	☐ ☐ ☐ ☐ ☐
48	No imagino otro recurso alternativo que sea mejor.	☐ ☐ ☐ ☐ ☐
49	Considero positivo la existencia de este recurso por los beneficios que proporciona y/o las oportunidades de uso futuro.	☐ ☐ ☐ ☐ ☐
50	El recurso es importante para alguien cercano (familiar, amigo, o conocido…).	☐ ☐ ☐ ☐ ☐
51	Este lugar o recurso forma parte de mi vida diaria.	☐ ☐ ☐ ☐ ☐
52	Disfruto el recurso estando con otras personas de mi entorno.	☐ ☐ ☐ ☐ ☐
53	La forma del recurso facilita su función.	☐ ☐ ☐ ☐ ☐
54	El recurso está bien organizado permitiendo que el total de la población haga uso de él (diseño universal).	☐ ☐ ☐ ☐ ☐
55	El recurso es suficientemente espacioso para el uso esperado o servicios a los que está destinado.	☐ ☐ ☐ ☐ ☐
56	El recurso tiene la infraestructura suficiente (instalaciones, medios materiales,…).	☐ ☐ ☐ ☐ ☐
57	Se observan elementos inspirados en espacios naturales (percepción visual de verde superior al 20% del espacio total del recurso).	☐ ☐ ☐ ☐ ☐
58	En general, este recurso es atractivo.	☐ ☐ ☐ ☐ ☐
59	La decoración es agradable (color, texturas,… mejoran el disfrute del recurso).	☐ ☐ ☐ ☐ ☐
60	El recurso es fácilmente adaptable a diferentes usos.	☐ ☐ ☐ ☐ ☐
61	El recurso ofrece una amplia disponibilidad horaria de sus servicios, o se adecua a necesidades específicas de sus usuarios.	☐ ☐ ☐ ☐ ☐
62	El recurso ofrece varios servicios o funciones a la vez.	☐ ☐ ☐ ☐ ☐
63	La forma del recurso es agradable o apacible y los usuarios se sienten cómodos en él.	☐ ☐ ☐ ☐ ☐
64	El recurso se ve cuidado, limpio y ordenado.	☐ ☐ ☐ ☐ ☐
65	El recurso es tranquilo, tiene baja exposición al ruido, o dispone de espacios que transmiten serenidad.	☐ ☐ ☐ ☐ ☐
66	Se respira un aire adecuado y los olores son agradables.	☐ ☐ ☐ ☐ ☐
67	La temperatura ambiental del recurso es adecuada para su uso.	☐ ☐ ☐ ☐ ☐
68	Hay suficiente luz natural en el lugar y su iluminación es adecuada.	☐ ☐ ☐ ☐ ☐
69	Existen mecanismos de protección y de seguridad (cámaras de vigilancia, cuerpos de seguridad…).	☐ ☐ ☐ ☐ ☐
70	La infraestructura y su diseño previenen el riesgo de lesiones.	☐ ☐ ☐ ☐ ☐
71	Existen pruebas de vandalismo en el recurso y su entorno (desperfectos…).	☐ ☐ ☐ ☐ ☐
72	Las normas de uso facilitan un espacio seguro.	☐ ☐ ☐ ☐ ☐
73	En el recurso o su entorno hay algún medio donde obtener ayuda de emergencia.	☐ ☐ ☐ ☐ ☐
74	Hay transparencia o campo visual entre el recurso y el exterior.	☐ ☐ ☐ ☐ ☐
75	Hay presencia ciudadana en el entorno durante el horario de utilización del recurso.	☐ ☐ ☐ ☐ ☐
76	El recurso, su infraestructura y diseño, transmiten confianza y seguridad para realizar las actividades esperadas.	☐ ☐ ☐ ☐ ☐
77	Tengo buenas referencias acerca del recurso (conocimiento, información positiva…).	☐ ☐ ☐ ☐ ☐
78	La edad no es determinante en la percepción de seguridad en el recurso o su entorno.	☐ ☐ ☐ ☐ ☐
79	Las diferencias de sexo/género no son determinantes en la percepción de seguridad en el recurso o su entorno.	☐ ☐ ☐ ☐ ☐
80	Las diferencias étnicas y culturales no son determinantes en la percepción de seguridad en el recurso o su entorno.	☐ ☐ ☐ ☐ ☐
81	La discapacidad no es determinante en la percepción de seguridad en el recurso o su entorno.	☐ ☐ ☐ ☐ ☐
82	El recurso presenta la cantidad y variedad de instalaciones u oferta de productos suficiente para prestar adecuadamente su función.	☐ ☐ ☐ ☐ ☐
83	Hay una oferta adecuada en el vecindario de este tipo de recurso.	☐ ☐ ☐ ☐ ☐
84	La utilización del recurso por un determinado usuario no limita la capacidad de ser usado por otras personas.	☐ ☐ ☐ ☐ ☐
85	Los criterios de acceso al recurso no discriminan a los potenciales usuarios del recurso.	☐ ☐ ☐ ☐ ☐
86	*Acerca de la privacidad*… El recurso permite el anonimato o cierto grado de intimidad cuando el usuario así lo requiere.	☐ ☐ ☐ ☐ ☐
87	El equipo humano que soporta el recurso propicia su durabilidad.	☐ ☐ ☐ ☐ ☐
88	En el recurso se observan medidas de innovación y mejora que aumentan su valor y repercuten positivamente en la salud.	☐ ☐ ☐ ☐ ☐
89	Con el paso del tiempo, el recurso muestra capacidad de adaptación estratégica a las nuevas necesidades de la comunidad.	☐ ☐ ☐ ☐ ☐
90	El recurso soporta bien su uso y desgaste, y requiere poco mantenimiento.	☐ ☐ ☐ ☐ ☐
91	El recurso es utilizado por un alto número de sus usuarios potenciales y de forma frecuente.	☐ ☐ ☐ ☐ ☐
92	Los beneficios que aporta el recurso a la comunidad son superiores a los costes.	☐ ☐ ☐ ☐ ☐
93	Estoy dispuesto a pagar por el uso del recurso un precio superior al actual para poderlo utilizar.	☐ ☐ ☐ ☐ ☐
94	Existen o pueden existir otros recursos sustitutivos para atender la misma función o funciones y de manera menos costosa para la comunidad.	☐ ☐ ☐ ☐ ☐
95	Considerando todos los aspectos, el recurso respeta el medio ambiente.	☐ ☐ ☐ ☐ ☐
96	El recurso promueve la participación de la población.	☐ ☐ ☐ ☐ ☐
97	El recurso es un claro “soporte” como receptor de numerosas actividades o servicios comunitarios.	☐ ☐ ☐ ☐ ☐
98	El recurso ejerce un papel proactivo en el desarrollo de actividades en su comunidad.	☐ ☐ ☐ ☐ ☐
99	El recurso es referente en la intermediación o enlace entre otras actividades o recursos.	☐ ☐ ☐ ☐ ☐
100	El recurso tiene relación directa con recursos influyentes o importantes de la comunidad.	☐ ☐ ☐ ☐ ☐
101	El recurso facilita las relaciones entre personas de la comunidad, contribuyendo al bienestar colectivo.	☐ ☐ ☐ ☐ ☐
102	La inclusión y participación social son relevantes en los objetivos y organización interna del recurso.	☐ ☐ ☐ ☐ ☐
103	El recurso contribuye a reducir las desigualdades sociales.	☐ ☐ ☐ ☐ ☐

The responses are of the 5-point Likert scale: “strongly agree” (5), “agree” (4), “neither agree nor disagree” (3), “disagree” (2), and “strongly disagree” (1). * The answers to this item correspond to the pair of adjectives: the positive adjective with a rating of 5 points and the negative adjective with a minimum rating of 1 point.
